# Supplementary material for: WordCommentsAnalyzer: A windows software tool for qualitative research
Source: F1000Res. 2018 Sep 4;7:536. Originally published 2018 May 3. [Version 2] doi: 10.12688/f1000research.14819.2 (PMC6137411; doi:10.12688/f1000research.14819.2)
Supplement: Supplementary file 3 [file f1000research-7-17614-s0002.tgz › b2a79496-4ada-47c4-9682-a99ce867836d.zip!/Eight Iranian Health Journals/Salmand_Iranian Journal of Ageing _25.docx]

© Salmand_Iranian Journal of Ageing . This work is licensed under CC BY-NC 4.0 (https://creativecommons.org/licenses/by-nc/4.0/).

Volume & Issue: دوره 7 - شماره 1

Title: رابطه حمایت اجتماعی و امیدواری با سلامت عمومی درسالمندان مرد مبتلا به دردهای مزمن جسمانی

http://salmandj.uswr.ac.ir/article-1-491-fa.html

اهداف: سالخوردگی مرحله‌ای از زندگی است که احتمال شیوع بیماری‌های جسمی را افزایش می‌دهد، به اذعان پژوهش‌ها، برخی از این بیماری‌ها مزمن می‌شوند و وضعیت روان شناختی سالمندان را نیز با خطر مواجه می‌کنند، ازجمله نیازمندی‌های این بیماران علاوه بر حمایت‌های پزشکی، فراهم ساختن کمک‌ها و توجهات روان‌شناختی نظیر حمایت اجتماعی و امید است که معمولاً وابسته به زمینه‌های محیطی است. مواد‌ و روش‌ها: با هدف بررسی رابطه حمایت اجتماعی و امیدواری با سلامت روان از ۸۴ سالمند مرد مبتلا به دردهای مزمن جسمانی (۵۰ نفر دارای همسر و ۳۴ نفر فاقد همسر با فاصله سنی ۶۹ تا ۸۸ سال) خواسته شد تا پرسشنامه‌های حمایت اجتماعی، امید و سلامت عمومی را تکمیل کنند. به دلایل فرهنگی در این تحقیق، تنها سالمندان مرد مورد بررسی قرارگرفتند. داده‌های بدست آمده با آزمون همبستگی پیرسون، تحلیل رگرسیون به شیوه گام به گام و آزمون t استیودنت برای گروه‌های مستقل با کمک نرم افزار SPSS نسخه ۱۶ مورد تجزیه‌وتحلیل قرارگرفت. یافته‌ها: نتایج نشان‌داد که بین حمایت اجتماعی، امید و سلامت عمومی سالمندان، رابطه معنادار وجود دارد (۰/۰۱>P). تحلیل رگرسیون به شیوه گام به گام نشان داد که متغیرهای امید و حمایت اجتماعی به ترتیب توانسته‌اند سلامت عمومی سالمندان مبتلا به دردهای مزمن را پیش‌بینی کنند (۰/۰۱>P). همچنین مقایسه میانگین متغیرهای سه گانه تحقیق (حمایت اجتماعی، امید و سلامت عمومی) در سالمندان واجد همسر و فاقد همسر نشان‌داد که این دو گروه از لحاظ این متغیرها با یکدیگر تفاوت معنادار دارند (۰/۰۱>P). سالمندان واجد همسر در هر سه متغیر در وضعیت مطلوب‌تر و مناسب‌تری به سر می‌برند. نتیجه‌گیری: هرچند کشور ما یکی از جوان‌ترین جمعیت را در اختیار دارد، بزودی دوره سالمندی را تجربه خواهدکرد و بسیار ضروری است که در برنامه‌ریزی‌های کلان اقتصادی، اجتماعی و فرهنگی به نیازهای اجتماعی و شبکه ارتباطی سالمندان توجه شود چراکه این موضوع می‌تواند اثرات مستقیمی بر سلامت و بهزیستی آنان داشته باشد. با توجه به نتیجه تحقیق حاضر، افزایش حس امید و برقراری حمایت‌های اجتماعی می‌تواند به ارتقای سلامت عمومی سالمندان مبتلا به دردهای مزمن منتهی شده، نهایتاً کیفیت زندگی مناسبی را برای آن‌ها فراهم آورد.

Title: بررسی تأثیر واقعیت‌درمانی گروهی بر سلامت روانی سالمندان مقیم آسایشگاه شهید هاشمی‌نژاد کهریزک

http://salmandj.uswr.ac.ir/article-1-484-fa.html

اهداف: هدف اصلی این پژوهش، تعیین تاثیر واقعیت درمانی گروهی بر سلامت روانی سالمندان مرد مقیم آسایشگاه شهید هاشمی نژاد کهریزک است. مواد و روش‌ها: طرح این پژوهش از نوع آزمایشی با پیش آزمون و پس آزمون و گروه کنترل بود. جامعه آماری در این تحقیق کلیه سالمندان مرد ساکن آسایشگاه شهید هاشمی نژاد کهریزک بودند. روش نمونه گیری در این تحقیق، روش در دسترس همراه با تصادفی ساده بود. ابتدا پرسشنامه سلامت عمومی روی 49 نفر از سالمندان (که مشکلات روانی و جسمانی حاد نداشتند) به صورت در دسترس اجرا شد. کسانی که نمره بالاتر از 22 را کسب کرده بودند، شناسایی شده و تعداد 30 نفر از آنها به طور تصادفی انتخاب شدند. سپس این تعداد در دو گروه 15 نفری (آزمایش و گواه) به طور تصادفی جایگزین شدند. گروه آزمایشی در 10 جلسه واقعیت درمانی گروهی شرکت داده شدند در حالی که گروه گواه هیچ گونه درمانی را دریافت نکردند. یافته‌ها: تحلیل نتایج با استفاده از آزمون آماری تحلیل کواریانس انجام شد که نشان داد واقعیت درمانی گروهی به طور معناداری (P<0/05) منجر به افزایش نمره کلی سلامت روانی، کاهش اضطراب و بهبود عملکرد اجتماعی سالمندان شده است ولی تاثیر آن بر افسردگی و نشانگان جسمانی سالمندان معنادار نبود. نتیجه‌گیری: تحلیل نتایج این مطالعه نشان داد که واقعیت درمانی گروهی در کاهش نشانگان اضطراب و بهبود عملکرد اجتماعی سالمندان تاثیر مثبت دارد و می‌تواند نمره کلی سلامت روانی آن‌ها را افزایش دهد. با برگزاری کارگاه‌های آموزشی برای کارکنان و پرستاران شاغل در آسایشگاه‌های سالمندان، این روش می‌تواند درمان مناسبی برای سالمندان مخصوصا در زمینه اضطراب و عملکرد اجتماعی باشد.

Title: وضعیت بهداشت فردی سالمندان روستاهای شهرستان مراوه‌تپه استان گلستان در سال 1389

http://salmandj.uswr.ac.ir/article-1-483-fa.html

اهداف: هدف اصلی این مطالعه بررسی وضعیت بهداشت فردی سالمندان روستاهای شهرستان مراوه‌تپه استان گلستان در سال 1389 به منظور استفاده از این اطلاعات در برنامه‌ریزی‌های بهداشتی برای ارتقاء سلامت سالمندان بود.  مواد و روش‌ها: دراین مطالعه توصیفی تحلیلی 119 نفر از سالمندان روستایی بالای 60 سال شهرستان مراوه تپه به روش نمونه گیری چند مرحله‌ای انتخاب شدند و داده‌ها از طریق پرسشنامه‌ای که روایی و پایایی آن مورد تأیید بود، با مراجعه به منزل و مصاحبه تکمیل شد. سپس داده‌ها وارد کامپیوتر شده و توسط نرم افرار SPSS 16  مورد تجزیه و تحلیل آماری قرار گرفت.  یافته‌ها: میانگین سنی سالمندان مورد مطالعه ۵/۶۴±۶۷/۵۳ سال بود. میانگین نمره رفتارهای ارتقادهنده سلامت سالمندان از پرسشنامه محقق ساخته 16±۶۰/۵۹ (از 100  نمره) بود. بین رفتارهای ارتقادهنده بهداشت فردی با سن، جنس، سطح تحصیلات، شغل و وضعیت زندگی با خانواده از نظر آماری ارتباط معنادار مشاهده نگردید ولی بین رفتارهای بهداشتی با وضعیت تأهل ارتباط معناداری وجود داشت.  نتیجه‌گیری: از آنجا که وضعیت رفتارهای ارتقادهنده سلامت سالمندان در سطح مطلوبی نمی‌باشد و با افزایش جمعیت سالمندان این اوضاع به مراتب نامطلوب‌تر و پیامدها و مشکلات آن بسیار چالش برانگیز خواهد شد لذا انجام مداخلات مقتضی در این زمینه به ویژه مداخلات آموزشی ضروری به نظر می‌رسد.

Title: مقایسه‌ کارایی تکالیف دوگانه در جوانان و سالمندان

http://salmandj.uswr.ac.ir/article-1-486-fa.html

اهداف: مطالعه‌ حاضر با هدف تعیین تأثیر سطح دشواری تکلیف تعادلی، بر کارایی تکالیف دوگانه در دو گروه جوانان و سالمندان، انجام گردیده است. مواد و روش‌ها: ۲۰ جوان (۳۰-۲۰ سال) و ۲۰ سالمند (۷۰-۶۵ سال) با روش نمونه‌گیری غیراحتمالی ساده در مطالعه شرکت کردند. تکالیف تعادلی، شامل ایستادن با پاهای مجاور هم روی سطح سخت و سطح فوم و تکلیف شناختی، زمان عکس العمل شنیداری بود. تکالیف تعادلی و تکلیف شناختی یک‌بار بصورت منفرد و یک‌بار بصورت همزمان انجام گردیدند. یافته‌ها: در گروه سالمندان؛ کارایی تکلیف تعادلی آسان در حالت منفرد و دوگانه تفاوت معنادار ندارد (P<0/05). نتیجه‌گیری: افزایش دشواری تکلیف وضعیتی می‌تواند به دلیل افزایش سطح بر انگیختگی، منجر به افزایش کارایی تکلیف وضعیتی در حالت دوگانه در مقایسه با تکلیف وضعیتی آسان در حالت دوگانه و نیز تکلیف وضعیتی آسان در حالت دوگانه شود.

Title: بررسی تأثیر تمرینات کششی ران و مچ پا در بهبود تعادل عملکردی سالمندان

http://salmandj.uswr.ac.ir/article-1-488-fa.html

اهداف: کاهش دامنه حرکتی حاصله از سفتی عضله و بافت همبند می‌تواند شیوع زمین‌ خوردن افراد سالمند را افزایش دهد. نتایج برخی تحقیقات نشان داده است که کاهش دامنه حرکتی ران و مچ پا یکی از علت‌های اصلی زمین ‌خوردن سالمندان به‌علت تأثیر آن روی الگوی راه رفتن می‌باشد. در این مطالعه تأثیر یک برنامه کششی ۸ هفته‌ای مفاصل ران و مچ پا روی بهبود تعادل سالمندان مورد بررسی قرار گرفت. مواد و روش‌ها: ۳۰ سالمند داوطلب بطور تصادفی به دو گروه کنترل و مداخله تقسیم شدند. شرکت‌کنندگان گروه مداخله یک برنامه کششی فلکسورهای ران و دورسی فلکسورهای مچ پا را سه بار در هفته برای ۸ هفته انجام دادند درحالی‌که گروه کنترل در طی این ۸ هفته فعالیت‌های روزمره خود را انجام می‌دادند. دامنه حرکتی ران و مچ پا با استفاده از یک گونیومتر استاندارد و میزان تعادل افراد مطابق با آزمون تعادلی Berg اندازه گرفته شد. تمامی شرکت‌کنندگان قبل و بعد از ۸ هفته مورد ارزیابی قرار می‌گرفتند. آزمون‌های آماری t مستقل و زوج به ترتیب جهت آنالیز بین و داخل گروهی استفاده گردید. یافته‌ها: در مقایسه با گروه کنترل، در شرکت‌کنندگان گروه مداخله انجام تمرینات کششی موجب گردید که دامنه حرکتی اکستنشن ران و دورسی فلکشن مچ پا به‌طور معناداری افزایش یابد همچنین این تمرینات باعث بهبود تعادل افراد گروه تمرین نیز گردید (۰/۰۰۱>P). نتیجه‌گیری: نتایج این تحقیق نشان داد که تمرینات کششی می‌تواند باعث بهبود دامنه حرکتی مفاصل و تعادل سالمندان گردد.

Title: وضعیت مرگ‌ومیر ناشی از تصادفات رانندگی در سالمندان ایرانی طی سال‌های 1385 الی 1387

http://salmandj.uswr.ac.ir/article-1-485-fa.html

اهداف: در سالمندان نرخ مرگ‌ومیر ناشی از تصادفات رانندگی بالاست. بررسی‌ها نشان می‌دهد که ‌20 درصد افراد سالمند، یک بار در سال در شهرها دچار حوادث ترافیکی می‌شوند. مواد و روش‌ها: این تحقیق از نوع مقطعی توصیفی می‌باشد. اطلاعات جمع‌آوری شده از مراکز پزشکی قانونی کل کشور در سال‌های 1385 الی 1387 جمع‌آوری و سپس تحلیل‌های لازم صورت گرفت. یافته‌ها: در مجموع تعداد 12029 مرگ به دنبال تصادفات رانندگی در افراد بالای 60 سال در این سال‌ها رخ داده است. به طور متوسط در هر سال %65 مرگ و میر در سنین جوانی-پیری و %34.5 در سنین پیری و حدود %0/5 در سنین پیری-پیری رخ داده است. به طور متوسط %19/2 متوفیان راننده، %56/9 را عابرین، %23/2 را سرنشینان تشکیل می‌دهند. همچنین در هر سال %46 مرگ و میر در حوادث درون شهری، %46/3 مرگ‌ومیر در حوادث برون شهری و %6/8 مرگ و میر در جاده‌های روستایی رخ داده است. نتیجه‌گیری: میزان حوادث جاده‌ای در ایران تقریباً 20 برابر بیشتر از سایر نقاط دنیاست. میزان مرگ‌ومیر در سال‌های 1385 تا 1387 در گروه سنی پیری و پیری-پیری با شیب ملایمی افزایش یافته و در گروه جوانی-پیری کاهش یافته است. فراوانی نسبی مرگ‌ومیر سالمندان طی سه سال مطالعه در جاده‌های برون‌شهری کاهش‌یافته اما در جاده‌های درون‌شهری افزایش پیدا کرده است.

Title: برآورد میزان شیوع افسردگی و عوامل فردی مرتبط با آن در سالمندان کرد مقیم منزل در سال ۱۳۸۹

http://salmandj.uswr.ac.ir/article-1-487-fa.html

اهداف: سالمندی بنا به آمار بدون شک یکی از مهم‌ترین مشکلات بهداشتی آینده جوامع خواهد بود. در این گروه سنی نیز مشکلات روانشناختی و به خصوص افسردگی از شایع‌ترین مشکلات می‌باشند. از دیگر سو با توجه به میزان شیوع متفاوت افسردگی در جوامع گوناگون به نظر می‌رسد که این بیماری می‌تواند در فرهنگ‌ها و قومیت‌های مختلف، دارای شیوعی متفاوت باشد. در این مطالعه میزان شیوع افسردگی و برخی عوامل فردی مرتبط با آن در سالمندان کرد بالای ۶۰ سال در سال ۱۳۸۹ مورد بررسی قرار گرفته است. مواد و روش‌ها: مطالعه اخیر یک مطالعه توصیفی مقطعی بوده که بر روی ۳۰۲ سالمند کرد بدون اختلال شناخت و مقیم جامعه پس از اخذ رضایت شرکت در تحقیق انجام شده است. در این پژوهش نمونه‌ها به‌صورت طبقه‌ای، تصادفی خوشه‌ای انتخاب شده و از یک پرسشنامه حاوی اطلاعات فردی و مقیاس افسردگی در سالمندان (GDS) نمونه ۱۵ سؤالی برای جمع آوری داده‌ها استفاده شده که به‌صورت مصاحبه با سالمند تکمیل شده است. اطلاعات حاصل توسط نرم افزار SPSS16 و با استفاده از روش‌های آماری تی تست، آنالیز واریانس و کای دو مورد تجزیه و تحلیل قرار گرفته است. یافته‌ها: بر اساس یافته‌های تحقیق میانگین سن افراد مورد مطالعه ۷۰/۶۹ سال (مردان ۷۲/۹۸ سال و زنان ۶۸/۲۶ سال) بوده و میانگین نمره افسردگی در سالمندان کرد ۵/۹۴ با انحراف معیار ۳/۴۰۵ بوده است. و همچنین میزان شیوع افسردگی خفیف ۳۸/۷درصد، افسردگی متوسط ۱۶/۳درصد و افسردگی شدید ۷درصد بوده است. در این تحقیق بین میانگین نمره افسردگی و جنس (۰/۰۳۲=P)، سن (۰/۰۲۳=P)، سطح تحصیلات (۰/۰۰۱>P) و همراه زندگی (۰/۰۰۶=P) ارتباط آماری معنادار مشاهده گردید و بین نمره افسردگی و محل سکونت در شهر و روستا، وضعیت تأهل و تعداد فرزندان ارتباط آماری معنادار وجود نداشت. نتیجه‌گیری: نتایج حاصل از مطالعه فوق بیانگر این مطلب است که افسردگی و به خصوص افسردگی خفیف از شیوع بالایی در میان سالمندان کرد برخوردار است لذا به نظر می‌رسد برنامه‌ریزی برای یافتن عوامل مرتبط با آن و انجام اقدامات مداخله‌ای می‌تواند در پیشگیری از پیشرفت آن به سمت اختلال افسردگی اساسی مؤثر باشد. مقایسه وضعیت سالمندان کرد با سایر قومیت‌ها پیشنهاد می‌گردد.

Title: سطح استقلال در فعالیت‌های روزمره جانبازان نابینای سالمند

http://salmandj.uswr.ac.ir/article-1-490-fa.html

اهداف: بررسی سطح استقلال جانبازان نابینای سالمند در اجرای فعالیت‌های روزمره، به منظورشناخت بهترجانبازان نابینا و هدایت برنامه‌ریزان جهت افزایش استقلال جانبازان نابینا در فعالیت‌های روزمره و بهبود کیفیت زندگی. مواد و روش‌ها: این مطالعه از نوع مقطعی‌ـ‌تحلیلی بود که بر روی ۳۱۲ نفر از جانبازان نابینای بالاتر از ۴۰ سال کشور در یک اردو در شهرستان مشهد صورت گرفت. به منظور درک بهتر از تغییرات زمان، همه آزمون‌ها در سه گروه سنی ۴۹-۴۰، ۵۹-۵۰ و بالای ۶۰ سال انجام و مقایسه شدند. برای ارزیابی فعالیت روزمره ‌ADL از اندیکس بارتل و برای IADL از ابزار Lawton-Bordy بهره‌ گرفته‌ شد. داده‌ها با انجام آزمون‌های ANOVA، پیرسون و جداول متقاطع تحلیل شدند. یافته‌ها: جانبازان نابینای بالای ۴۰ سال مورد مطالعه، میانگین سنی ۴۷/۳ سال داشتند که ٪۹۹ آن‌ها را مردان تشکیل می‌دادند. همه افراد مورد مطالعه متأهل بودند و ٪۸۲ آن‌ها فاقد شغل بودند. اغلب این افراد را گروه با تحصیلات دانشگاهی تشکیل می‌دادند. همه افراد مورد مطالعه، در حداقل یک فعالیت دچار وابستگی بودند. رابطه بین تحصیلات و برخی از فعالیت‌های روزمره مانند غذاخوردن، حرکت در سطوح غیرهم‌سطح از نظر آماری معنادار بود. جانبازان نابینای بالای ۶۰ سال در استفاده از تلفن، مدیریت دارو، مدیریت مالی، حرکت در سطوح غیرهمسطح و استفاده از پلّه وابستگی قابل ملاحظه‌ای در مقایسه با گروه‌های زیر ۵۰ سال داشتند. نتیجه‌گیری: کاسته‌شدن از سطح استقلال فردی در فعالیت های روزمره عموماً با افزایش سن در همه آحاد جامعه رخ می‌دهد ولی سن بروز وابستگی‌ها اهمیت دارد که به نظر می‌رسد در افراد نابینا زودتر از زمان مورد انتظار رخ می‌دهد. لذا ممکن است، برخی از فعالیت‌ها با آموزش و انجام شیوه‌های توانبخشی به تأخیر افتاده و موجب استقلال بیشتر آنان گردد، به ویژه که فعالیت‌هایی مانند استفاده از تلفن، تحرک و جابجایی در ایجاد ارتباطات اجتماعی کمک می‌کنند. مطالعات بیشتر برای شناسایی و تأثیر این روش‌ها بر روی عملکرد و استقلال سالمندان توصیه می‌شود.
